# Predicting the potential geographical distribution of mango, an important tropical economic tree species, under current and climate change based on Maxent model

**DOI:** 10.3389/fpls.2025.1633654

**Published:** 2025-08-06

**Authors:** Sangui Yi, Yuanhe Huang, Zongling Liu, Zhengjie Zhu, Hongxin Su

**Affiliations:** ^1^ School of Basic Medical Sciences, Youjiang Medical University for Nationalities, Baise, China; ^2^ Guangxi Key Laboratory of Biology for Mango, Baise University, Baise, China; ^3^ Key Laboratory of Environment Change and Resources Use in Beibu Gulf, Ministry of Education, Nanning Normal University, Nanning, China; ^4^ Guangxi Key Laboratory of Earth Surface Processes and Intelligent Simulation, Nanning Normal University, Nanning, China

**Keywords:** mango, climate change, Maxent model, potential suitable habitat, climate factor

## Abstract

**Introduction:**

Mango is a major tropical economic tree species in China, along with being a vital source of livelihood for farmers and an important maintainer of ecosystem services in southern China. Identifying the potential suitable habitats for mango under current and future climate scenarios, along with key influencing factors, can inform mango plantation. However, little is known about these.

**Methods:**

Using Maxent, we modeled the current and future potential suitable habitats for mango, evaluated the impact of environmental variables on their distribution, and identified shifts related to climate change in their distribution.

**Results:**

The results showed that the current potential suitable habitats for mango were primarily located in southern China, within the tropical and subtropical regions. Under climate scenarios of both SSP585 and SSP126, the potential suitable habitats not only encompassed the southern provinces of China that were already covered but also expanded northward to include central provinces, particularly Sichuan and Chongqing municipalities. Mango exhibited a clear tendency to migrate toward higher altitudes and latitudes under SSP585 scenario, whereas the trend of mango migration to such areas was less pronounced under SSP126 scenario. Mean Temperature of Coldest Quarter, Annual accumulated temperature (≥10°C), Precipitation of Coldest Quarter, and UV-B Seasonality were identified as the main factors shaping the distribution of the potential suitable habitats for mango.

**Discussion:**

Our recommendation to adapt to climate change is to expand mango cultivation to high-latitude/altitude areas, particularly Sichuan-Chongqing in central China, along with water-saving irrigation, shade management, development of drought- and disease-resistant cultivars, and mapping of the potential suitable habitats for different varieties.

## 1 Introduction

Climate is the main factor affecting the population size and physiological metabolism of species, as well as an important factor affecting species distribution (Bedair et al., 2025; Wiens and Zelinka, 2024; Lawler et al., 2009). The Sixth Assessment Report of the Intergovernmental Panel on Climate Change (IPCC) states that since 2011, greenhouse gases in the atmosphere have continued to increase, and the atmosphere, land, and ocean on the Earth’s surface have continued to warm (IPCC, 2023). Agricultural ecosystems are sensitive to climate change (Zhang et al., 2017). It can change the planting structure and distribution of crops and even exacerbate meteorological disasters in some areas, making crop production unstable and posing huge challenges to agricultural production (Tovar et al., 2022; Zhang et al., 2017; Urban et al., 2013). Therefore, predicting the potential geographical distribution of crops under current and climate scenarios, along with the influencing factors, is crucial for agricultural planning and management to adapt to climate change.

Mango (*Mangifera indica L*) is a tropical evergreen large tree with early fruiting and high yield, resulting in high planting efficiency (Jahurul et al., 2015). As one of the world’s top five tropical fruits, its fruit has become widely loved by consumers due to its delicious taste and rich vitamins. So, it is known as “the king of fruits” (Baradevanal et al., 2021; Tharanathan et al., 2006). Originating from the border region between India and Myanmar (Bally, 2006; Tharanathan et al., 2006), mango is now widely distributed primarily in tropical and subtropical areas, and the mango industry has emerged as one of the most important tropical crop industries globally (Guo et al., 2023; Bally, 2006). After years of introduction, trial planting, and expansion, it has developed into a significant tropical economic fruit in China, holding an important role in agricultural production in the southern region. In 2020, China ranked third in the world for mango cultivation, trailing only behind India and Thailand (Guo et al., 2023).

The optimal conditions for mango growth include an annual average temperature ranging from 21 to 27°C, a minimum average temperature of 12°C in the coldest month, an annual precipitation exceeding 1300 mm (or adequate irrigation in the absence of sufficient rainfall), a three-month dry period during the flowering stage, ample sunlight exposure, and well-drained soil (Liu, 2017). This suggests that climate factors fundamentally constrain its potential suitable habitats (Baradevanal et al., 2021; Sthapit et al., 2012). Although global warming may theoretically expand potential suitable habitats for mango due to the species’ preference for warm humid climates, the nonlinear effects of extreme temperatures create substantial uncertainty in predicting potential suitable habitats in the future (Baradevanal et al., 2021; Sthapit et al., 2012). However, many studies on mango mainly focused on disease and pest management (Baradevanal et al., 2021), phytochemical composition (Jahurul et al., 2015; Tharanathan et al., 2006), yield optimization (Fukuda et al., 2013), and post-harvest processing technologies (Liu et al., 2023; Owino and Ambuko, 2021; Tharanathan et al., 2006). There is relatively little research on the key factors and the potential suitable habitats for mango under climate change.

Species distribution models (SDMs) infer species’ ecological needs and potential distributions by correlating observed occurrence data with environmental variables (Araújo and Peterson, 2012; Elith and Leathwick, 2009). Initially developed in the 1970s for crop prediction, SDMs gained prominence in the 21st century with advances in computing, GIS, and open environmental data (Guisan and Thuiller, 2005; Austin, 1971). Various methods have emerged, including GARP, Maxent, ENFA, GAM, CART, and ANN (Yi et al., 2020; Zhang et al., 2018; Townsend Peterson et al., 2007; Heikkinen et al., 2006; Hirzel et al., 2002). Among these, Maxent is widely used due to its robustness to sample bias, minimal data requirements, and open-source accessibility (Phillips et al., 2017; Phillips and Dudík, 2008; Tsoar et al., 2007; Hernandez et al., 2006; Elith et al., 2006), making it a key tool in ecology, biogeography, and agronomy (Zhao et al., 2024; Phillips et al., 2006; Phillips and Dudík, 2008).

To evaluate the distribution of the potential suitable habitats for mango and the key factors affecting suitability, we used the Maxent method to predict its potential suitable habitats in China based on a large number of geo-referenced records and recent surveys (Kang et al., 2025; Zhao et al., 2024). The objectives of this study were to (1) identify the important environmental factors that affect the potential suitable habitats for mango, (2) examine the potential suitable habitats for mango under current and climate scenarios, and (3) quantify the pattern changes in the potential suitable habitats for mango under future climate conditions, and provide the theoretical basis for their cultivation.

## 2 Materials and methods

**Figure 1:**
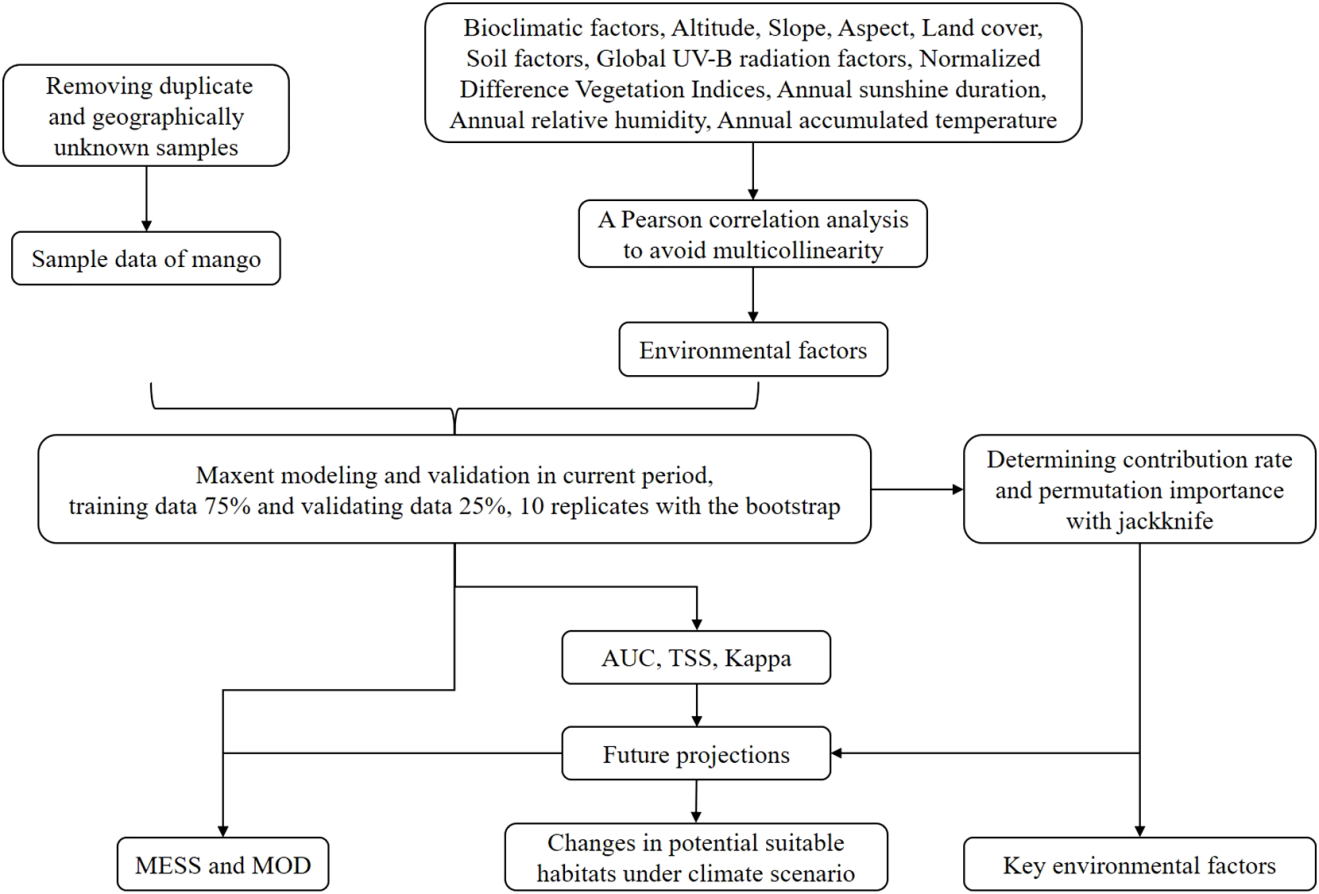
Research flow chart.

The research method is summarized as shown in **Figure 1**.

### 2.1 Sample data

**Figure 2:**
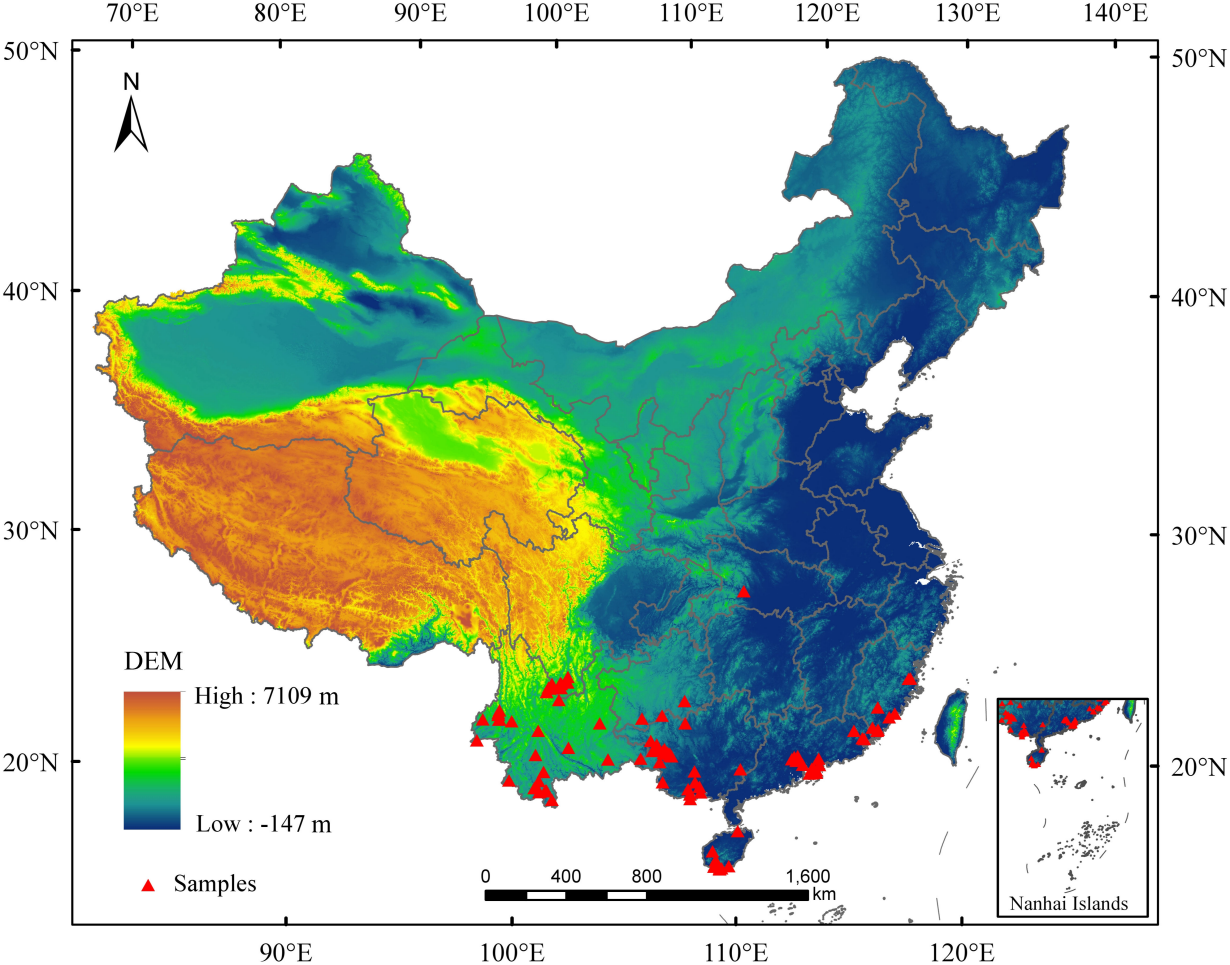
The distribution sample points of mango in China.

Mango distribution data were collected from field surveys, published literature, the Chinese Virtual Herbarium (https://www.cvh.ac.cn/), and the Global Biodiversity Information Facility (https://www.gbif.org/). To enhance prediction accuracy, duplicate and location-uncertain records were excluded, retaining only one sample per 2.5’ × 2.5’ grid (Zhao et al., 2021). Finally, 109 occurrence points were used for modeling (**Figure 2**).

### 2.2 Environmental factors

Mango is highly sensitive to temperature, precipitation, and their seasonal variations. The bioclimatic variables directly reflect the thermal and water conditions required for its growth, flowering, and fruiting. Altitude, slope, and aspect affect local microclimates, while land cover indicates habitat suitability and competition. Soil properties impact root development, nutrient uptake, and water retention. UV-B radiation affects flowering and disease susceptibility, NDVI reflects vegetation health, and sunshine duration influences photosynthesis and sugar accumulation. High humidity increases fungal disease risks (e.g., anthracnose), and accumulated temperature determines growing season length and heat requirements for fruiting. These factors collectively define mango’s ecological constraints (Bedair et al., 2025; Kang et al., 2025; Zhao et al., 2021; Zhang et al., 2018; Liu, 2017). Therefore, 51 environmental factors influencing mango distribution were selected, including 19 bioclimatic factors (2.5’) from the World Climate Database (Fick and Hijmans, 2017), Digital Elevation Model (250 m) from the Geospatial Data Cloud (http://www.gscloud.cn/), land cover indices (30”) from the Global Map data archives (https://globalmaps.github.io/), 16 soil factors (0–20 cm) from the Harmonized World Soil Database (https://www.fao.org/soils-portal/data-hub/soil-maps-and-databases/harmonized-world-soil-database-v20/en/), UV-B1–6 radiation (15’) from the gIUV database (https://www.ufz.de/gluv/index.php?en=32435), The Normalized Difference Vegetation Indices (30”) from the Geographical Information Monitoring Cloud Platform (http://www.dsac.cn/), and three climatic factors (annual sunshine duration, annual relative humidity, and annual accumulated temperature (≥ 10°C, ≥ 5°C, and ≥ 0°C); 1 km) from the Resource and Environmental Science Data Platform (https://www.resdc.cn/DOI/DOI.aspx?DOIID=96). Based on the availability of data and references to other literature, we believe environmental factors other than bioclimatic factors will not undergo significant changes in this study (Hill et al., 2024; Zhang et al., 2018). Therefore, the current projection (1970-2000) included sample data, bioclimatic factors, NDVI, and climatic factors from 1970 to 2000, whereas DEM and land cover were from 2003, soil factors from 2023, and UV-B radiations from 2004 to 2013. Future projections (2041-2060, 2061-2080, and 2081-2100) employed updated bioclimatic components for each period, but other factors remained constant. We used BCC-CSM2-MR model data from CMIP6 under SSP126 (low emission, 2.6 W/m² radiative forcing – by 2100) and SSP585 (high emission, 8.5 W/m² by 2100) scenarios (Zhao et al., 2021).

All environmental factors were resampled to a 2.5’ resolution. To prevent multicollinearity, Pearson correlation analysis (SPSS 20.0) identified highly correlated variables (|R| >0.75), from which those with higher contribution rates and permutation importance (Santana et al., 2019) were retained for Maxent modeling, resulting in 16 final variables (**Table 1**).

**Table 1:** The percentage contribution and the permutation importance of the environmental factors included in the Maxent models.

Symbol	Environmental factors	Contribution rate (%)	Permutation importance (%)
Bio11	Mean Temperature of Coldest Quarter	71.3	87.3
TEMJW10	Annual accumulated temperature (≥ 10°C)	6.2	2.2
Bio19	Precipitation of Coldest Quarter	4	1.5
NDVI	Normalized Difference Vegetation Index	2.7	0.7
UVB4	Mean UV-B of Lowest Month	2.3	0.4
UVB2	UV-B Seasonality	2.3	1.6
Elev	Elevation	1.7	1.6
RHU	Annual relative humidity	1.6	0.9
LC	Land cover	1.5	0.7
SSD	Annual sunshine duration	1.3	0.4
Bio3	Isothermality	1.2	0.7
TEXTURE_USDA	Soil texture class	1.1	0.2
Bio13	Precipitation of Wettest Month	1	0.6
CEC_CLAY	Cation exchange capacity of the clay fraction	0.9	0.5
Slope	Slope	0.7	0.4
Aspect	Aspect	0.5	0.2

### 2.3 Simulations of the potential suitable habitats

The SDMs were established with Maxent 3.4.4 (https://biodiversityinformatics.amnh.org/open_source/maxent/). 75% of the distribution sample data were used to train, and the remaining 25% were used to validate the model. 10 replicates were performed with the bootstrap in this study. The jackknife method was used to determine the contribution rate and permutation importance of all environmental factors.

Kuenm package in R 4.2.3 was used to optimize two parameters, including regularization multiplier (RM) and feature class (FC). In all, 1240 candidate models, with parameters reflecting all combinations of 40 RM settings (from 0.1 to 4 with the interval of 0.1) and 29 FC combinations, have been evaluated. All candidate models were selected based on statistical significance (Partial receiver operating characteristic (ROC), omission rates (OR), and the Akaike information criterion (AICc)). Initially, candidate models were selected and retained only those with statistical significance; next, models were further refined with the OR criterion, aiming for an OR of less than 5% whenever feasible; finally, models with the lowest delta AICc (less than 2) were selected from the pool of significant models that also met the low-OR criteria.

To assess model performance, we used the area under the ROC curve (AUC), true skill statistic (TSS), and KAPPA (Yi et al., 2021; Zhao et al., 2021; Allouche et al., 2006). AUC values indicate performance levels: 0.90-1.00 (excellent), 0.80-0.90 (good), 0.70-0.80 (fair), 0.60-0.70 (poor), and 0.50-0.60 (failure). TSS and KAPPA, ranging from -1 to +1, account for omission and misclassification errors, with values closer to +1 indicating better performance and values below 0 suggesting poor accuracy. These metrics provide a comprehensive evaluation of the model.

The species distribution map (ranging from 0 to 1) showed occurrence probability (P), and areas were identified as unsuitable (P < 0.0964) or suitable (P > 0.0964) based on the maximum test sensitivity plus specificity logistic threshold (MTSPS) (Zhang et al., 2022).

After modeling the current potential suitable habitats, projections were made under climate scenarios to predict how the potential suitable habitats would change (expansion, contraction, and stability).

### 2.4 The core distributional shifts

The trends of change in the potential suitable habitats were calculated, and the centroids for current and future habitats were compared using the SDM toolbox (Zhang et al., 2018). The core shifts in the distribution of mango were summarized. This process involved condensing the distribution of mango into a single centroid point, which allowed for the creation of a vector file. This file visually represented the magnitude and direction of the predicted changes over time. Finally, the shifts in distribution were examined by tracking how the centroid varied with SDMs.

### 2.5 The multivariate environmental similarity surface and the most dissimilar variable analysis

The MESS and MoD were used to evaluate climate anomalies and identify key drivers of the potential suitable habitat changes, respectively. MESS measures similarity (S) between reference and projected climates, where S>0 indicates higher similarity (maximum at S=100), while S<0 reflects climate anomalies, with more negative values indicating greater deviations beyond reference ranges. MoD pinpoints the least similar variable at each point, highlighting potential key distributional drivers. Analyses were performed using the “density. tools. Novel” tool in maxent.jar file (Zhao et al., 2021).

## 3 Results

### 3.1 The species distribution model and its accuracy

**Figure 3:**
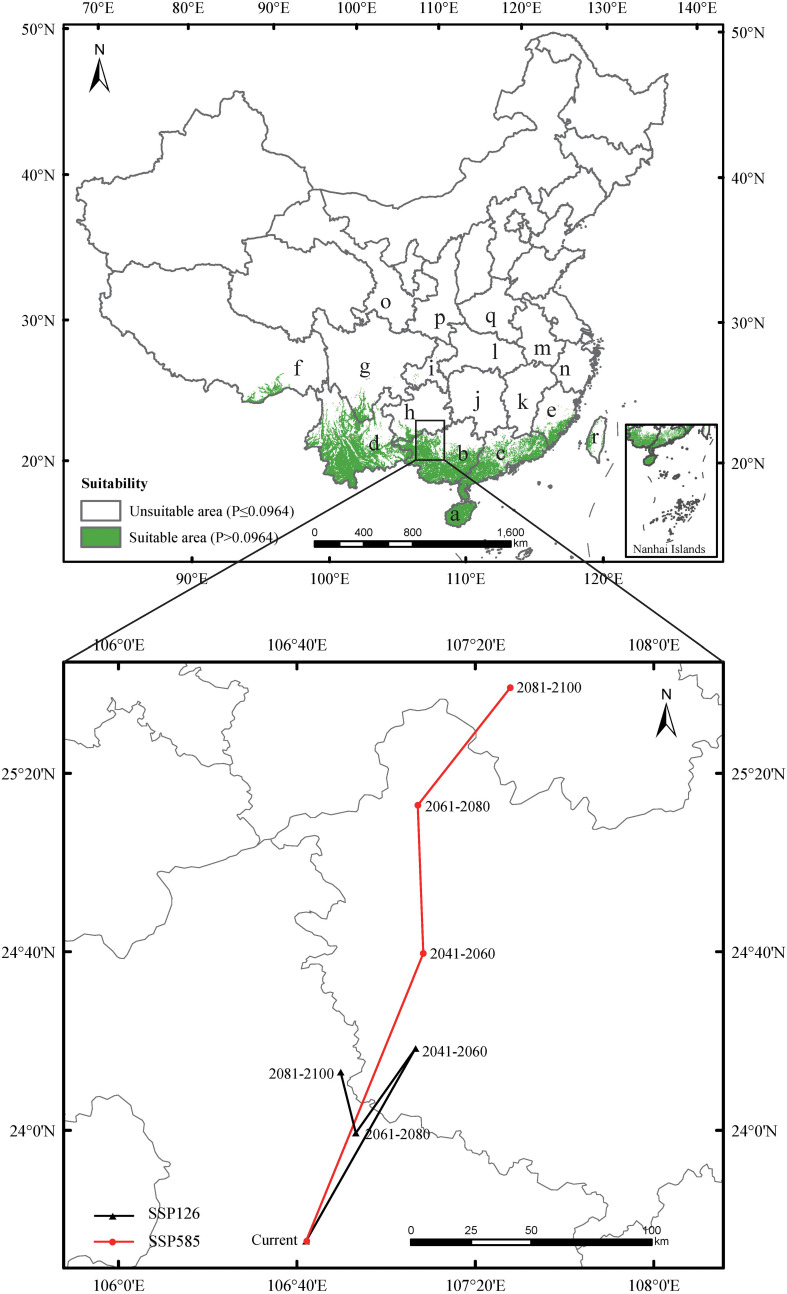
Predicted the current distribution and the core distributional shifts under different climate scenarios. The arrow indicates the magnitude and direction of predicted change through time. The locations were marked as follows: a - Hainan, b - Guangxi, c - Guangdong, d - Yunnan, e - Fujian, f - Xizang, g - Sichuan, h - Guizhou, i - Chongqing, j - Hunan, k - Jiangxi, l - Hubei, m - Anhui, n - Zhejiang, o - Ningxia, p - Shanxi, q - Henan, r – Taiwan.

The AUC, TSS, and KAPPA values were 0.989, 0.910, and 0.870, respectively, indicating strong model performance. The current potential suitable habitat for mango covered approximately 4.81×105 km², primarily located in Hainan, Guangdong, Guangxi, Yunnan, southern Tibet, southern Sichuan, southern Guizhou, southeast Fujian, and western Taiwan. Additionally, a sporadic distribution occurred in Chongqing (**Figure 3**).

### 3.2 Key environmental factors

**Figure 4:**
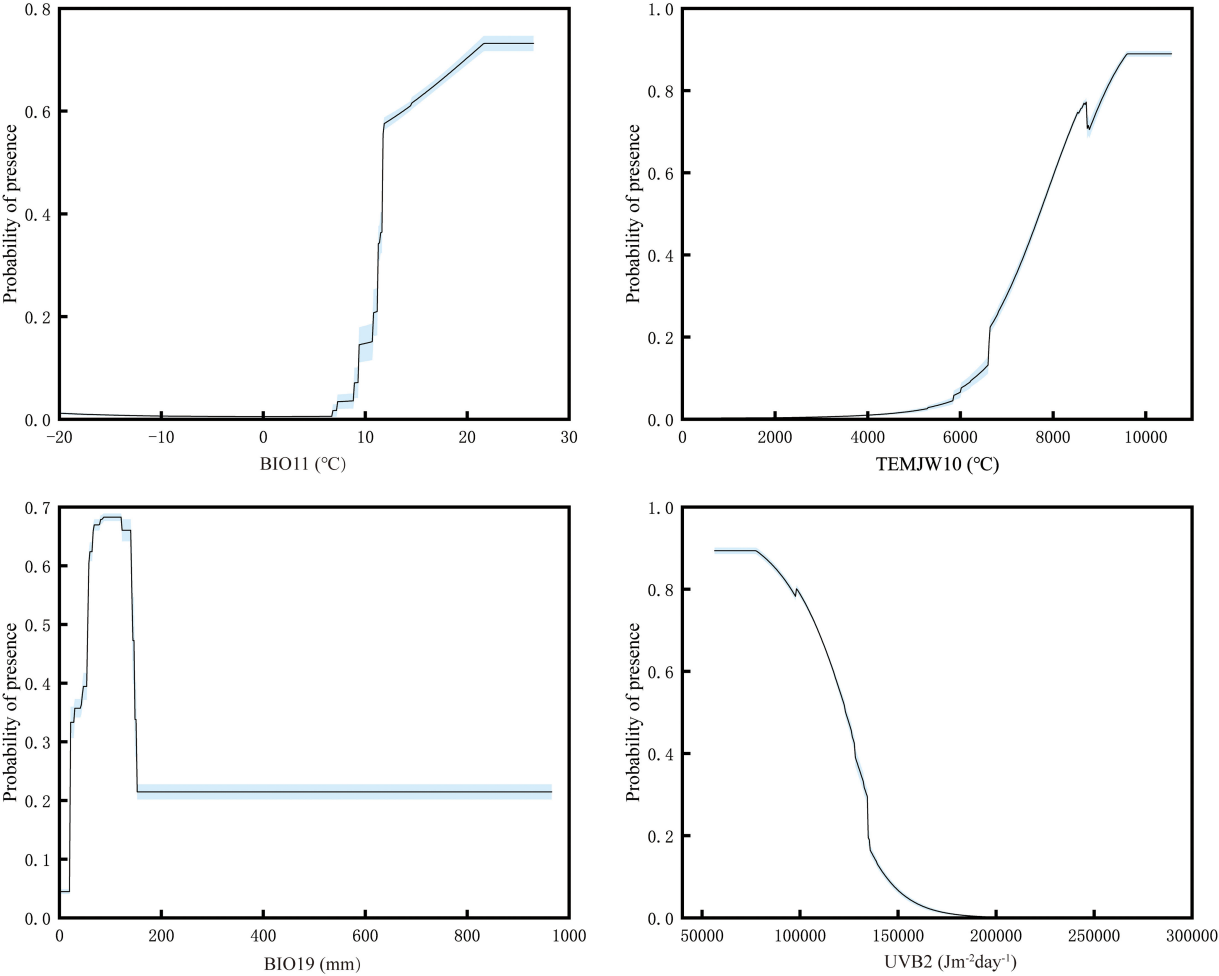
Response curves for important environmental factors in the species distribution model. Factors abbreviations were as follows: Bio11 - Mean Temperature of Coldest Quarter, TEMJW10 - Annual accumulated temperature (≥ 10°C), Bio19 - Precipitation of Coldest Quarter, UVB2 - UV-B Seasonality.

The jackknife test showed that mean temperature of coldest quarter (Bio11; contribution rate 71.3% and permutation importance 87.3%), annual accumulated temperature (≥ 10°C) (TEMJW10; contribution rate 6.2% and permutation importance 2.2%), precipitation of coldest quarter (Bio19; contribution rate 4% and permutation importance 1.5%), and UV-B seasonality (UVB2; contribution rate 2.3% and permutation importance 1.6%) were the main factors affecting the current potential suitable habitats for mango. Their cumulative contribution rate and permutation importance reached 83.8% and 92.6%, respectively (**Table 1**). These high cumulative values suggest that these four factors play a dominant role in shaping the potential suitable habitats of mango, with mean temperature of the coldest quarter being the most influential due to its significantly higher individual contribution rate and permutation importance compared to the other factors. Response curves of important factors were presented (**Figure 4**). Occurrence probability increased with rising Bio11 and TEMJW10 but decreased with elevated UVB2. For Bio19, we observed an unimodal response, with probability peaking at intermediate values. The suitable range of Bio11 was greater than 9.29°C. This aligns with mango’s tropical and subtropical origin, as it requires relatively warm conditions even in the coldest quarter to avoid cold damage. The suitable range of TEMJW10 was greater than 6249.63°C. This reflects mango’s high temperature demand during the growing season, as sufficient accumulated temperature is necessary for fruit ripening. The suitable range of Bio19 was greater than 19.67 mm, and the occurrence probability peaked at 0.68 as Bio19 approached 80.78 mm. This indicates that while a certain amount of precipitation in the coldest quarter is beneficial for mango, excessive or insufficient precipitation may reduce its suitability. The suitable range of UVB2 was less than 144472.61 Jm^-2^day^-1^. This suggests that mango is sensitive to high levels of UV-B radiation seasonality, which may have adverse effects on its growth and development.

### 3.3 Changes of the potential suitable habitats under climate scenarios

**Figure 5:**
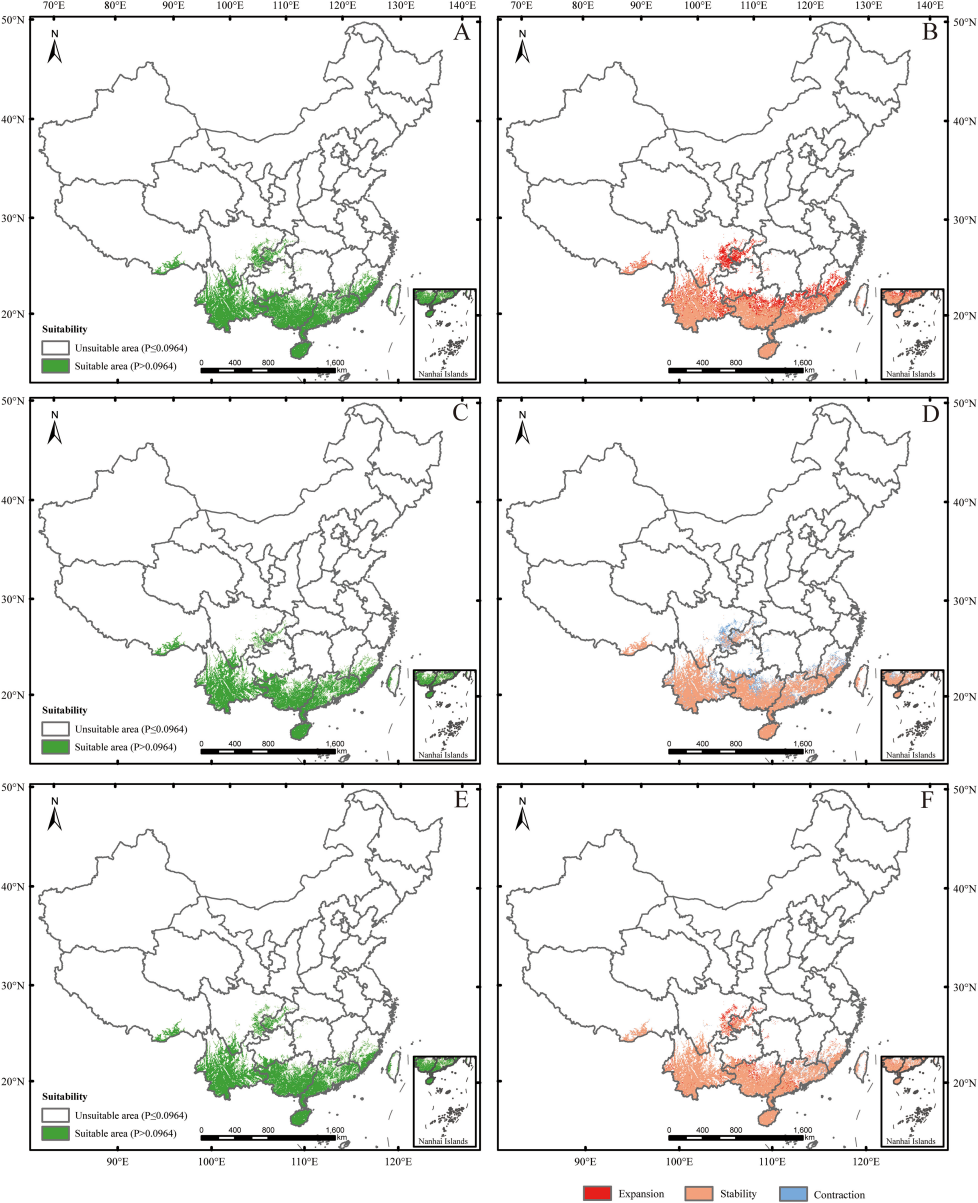
Future species distribution models (SDMs) under SSP126 scenario. **(A, C, E)** represent SDMs for the periods 2041-2060, 2061-2080, and 2081-2100, respectively; **(B, D, F)** represent comparisons of SDMs between the current period and 2041-2060, between 2041–2060 and 2061-2080, and between 2061–2080 and 2081-2100, respectively. The locations were marked the same as in **Figure 3**.

Under SSP126 (2041-2060) scenario, suitable areas would be primarily increased in eastern Sichuan, central western Chongqing, central Zhejiang, southern Jiangxi, northern Guangdong, northern Guangxi, southern Guizhou, southern Xizang, and eastern Yunan (**Figures 5A, B**; **Table 2**), totaling approximately 242,771.84 km². The decreased suitable areas were approximately 557.46 km² and located primarily in Taiwan (**Figures 5A, B**; **Table 2**). Under SSP126 (2061-2080) scenario, the changes remained largely opposite to those observed in 2041-2060, with suitable areas decreasing from 7.37% to 6.23% and unsuitable areas increasing from 92.63% to 93.77% (**Figures 5C, D**; **Table 3**). Under SSP126 (2081-2100) scenario, suitable areas would be primarily increased in eastern Sichuan, central western Chongqing, central Guangdong, and north Guangxi, totaling approximately 59,774.42 km2 (**Figures 5E, F**; **Table 2**). The decreased suitable areas were approximately 11,503.85 km² and located primarily in parts of Fujian, Guangdong, Jiangxi, Taiwan, and Xizang (**Figures 5E, F**; **Table 2**). The centroid of the current potential suitable habitat was located at the position of 106° 41’ E and 23° 36’ N in western Guangxi, with an altitude of 689 m. And it shifted northward to 24° 18’ N and 107° 6’ E with an altitude of 634 m under SSP126 (2041-2060) scenario, then shifted southward to 23° 59’ N and 106° 53’ E with an altitude of 721 m under SSP126 (2061-2080) scenario, and continued to shift northward to 24° 13’ N and 107° 7’ E with an altitude of 606 m under SSP126 (2081-2100) scenario (**Figure 3**).

**Table 2:** Dynamics of changes in distribution area under future climate scenarios.

Periods	Area (km^2^)		
	Expansion	Stability	Contraction
SSP126 (1970-2000)-(2041-2060)	242771.84	525199.03	557.46
SSP126 (2041-2060)-(2061-2080)	1647.03	649156.77	118814.01
SSP126 (2061-2080)-(2081-2100)	59774.42	639299.95	11503.85
SSP585 (1970-2000)-(2041-2060)	326668.88	525604.45	152.03
SSP585 (2041-2060)-(2061-2080)	171518.88	841909.63	10363.60
SSP585 (2061-2080)-(2081-2100)	190801.77	1005218.72	8209.80

**Table 3:** The potential distribution areas and their proportions under current and future climate scenarios.

Periods	Area (km^2^)		Portion of area (%)	
	Unsuitable	Suitable	Unsuitable	Suitable
Current	9118918.60	481081.40	94.99	5.01
SSP126 2041-2060	8892470.29	707529.71	92.63	7.37
SSP126 2061-2080	9002101.73	597898.27	93.77	6.23
SSP126 2081-2100	8956061.20	643938.80	93.29	6.71
SSP585 2041-2060	8812873.87	787126.13	91.80	8.20
SSP585 2061-2080	8658890.45	941109.55	90.20	9.80
SSP585 2081-2100	8486189.04	1113810.96	88.40	11.60

**Figure 6:**
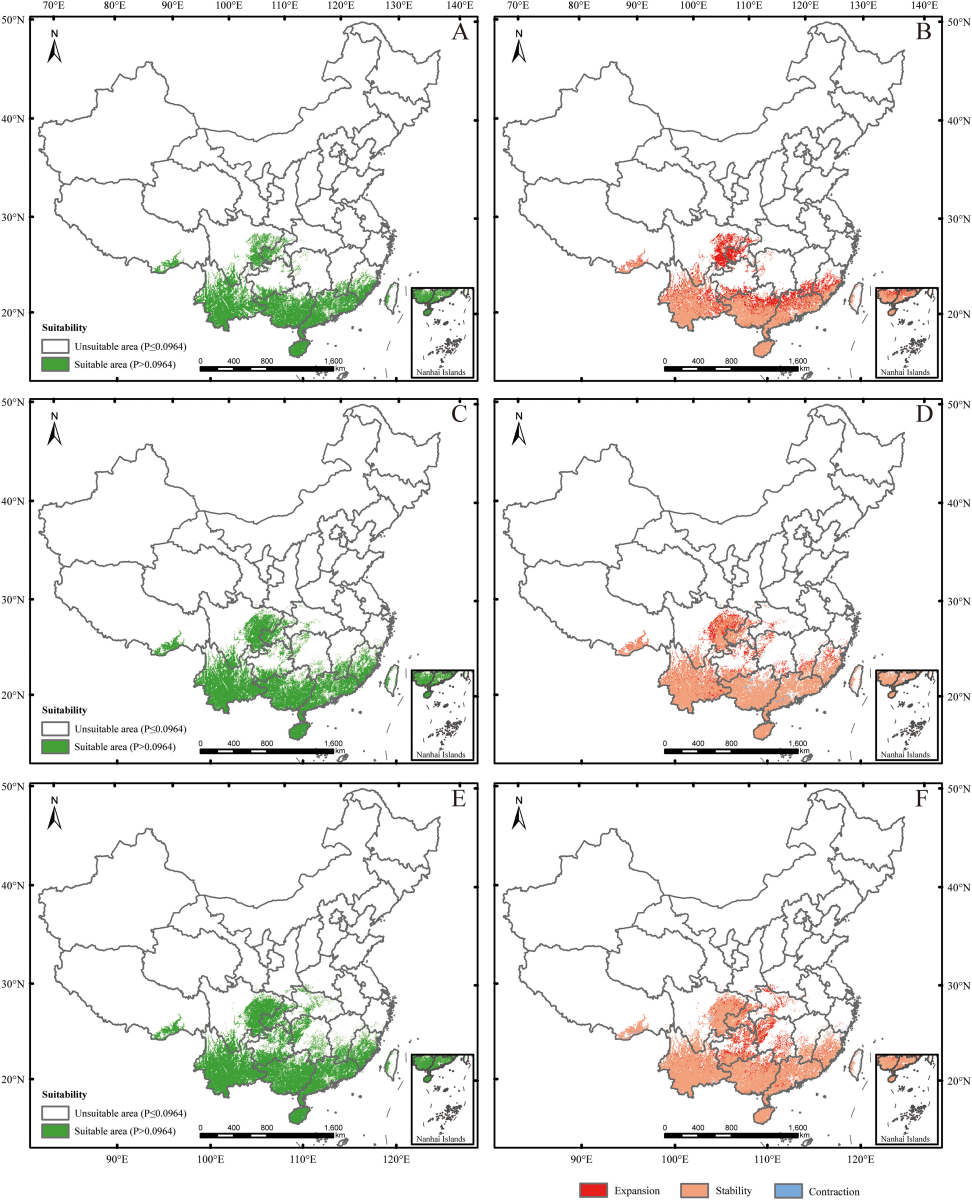
Future species distribution models (SDMs) under SSP585 scenario. **(A, C, E)** represent SDMs for the periods 2041-2060, 2061-2080, and 2081-2100, respectively; **(B, D, F)** represent comparisons of SDMs between the current period and 2041-2060, between 2041–2060 and 2061-2080, and between 2061–2080 and 2081-2100, respectively. The locations were marked the same as in **Figure 3**.

Under SSP585 (2041-2060) scenario, the changes in the potential suitable habitats were similar to those under SSP126 (2041-2060) scenario, with the suitable area expanding by 3.19% (**Figures 6A, B**; **Tables 2**, **3**). Under SSP585 (2061-2080) scenario, suitable areas would be primarily increased in eastern Sichuan, central western Chongqing, northern Zhejiang, southern Jiangsu, central southern Jiangxi, southern and northern Hunan, southern Hubei, southern and northern Guizhou, Yunnan, and southern Xizang, totaling approximately 171,518.88 km² (**Figures 6C, D**; **Table 2**). The decreased suitable areas were approximately 10,363.60 km² and primarily located in Guangdong and Guangxi (**Figures 6C, D**; **Table 2**). Under SSP585 (2081-2100) scenario, suitable habitats would primarily expand northward, including Guizhou, Hunan, Hubei, and northern Guangxi, totaling approximately 190,801.77 km² (**Figures 6E, F**; **Table 2**). The decreased suitable areas were approximately 8,209.80 km² and primarily located in Jiangsu, Zhejiang, Sichuan, Yunnan, Xizang, and Taiwan (**Figures 6E, F**; **Table 2**). The centroid of the potential suitable habitat shifted northward to 24° 39’ N and 107° 8’ E with an altitude of 683 m under SSP585 (2041-2060) scenario, shifted northward to 25° 12’ N and 107° 7’ E with an altitude of 733 m under SSP585 (2061-2080) scenario, and continually shifted northward to 25° 38’ N and 107° 27’ E with an altitude of 1,023 m under SSP585 (2081-2100) scenario (**Figure 3**).

Overall, under both climate scenarios, most potential suitable habitats were concentrated in southern provinces, with some in central provinces (**Figures 5A, C, E**, **6A, C, E**), which aligns with the species’ preference for warm and humid climates typical of these regions. The potential suitable habitat areas increased under both scenarios, with the highest expansion under SSP585 scenario (**Tables 2**, **3**), reflecting the stronger climate forcing effect of high-emission pathways on habitat range dynamics. Under SSP126 scenario, the potential suitable habitats initially expanded northward (2041-2060), then contracted southward (2061-2080), before slightly expanding north again (2081-2100) (**Figure 5**; **Tables 2**, **3**), a pattern that may be associated with intermittent climate stabilization efforts under the low-emission scenario. In contrast, SSP585 exhibited continuous northward expansion across all periods (**Figure 6**; **Tables 2**, **3**), driven by unabated temperature rises that consistently push suitable habitats poleward. Core distribution shifts under SSP585 trended toward higher altitudes/latitudes, while no clear pattern emerged under SSP126 (**Figure 3**), highlighting the differential impact of emission trajectories on the species’ habitat migration patterns.

### 3.4 Analysis of the MESS and MoD

**Figure 7:**
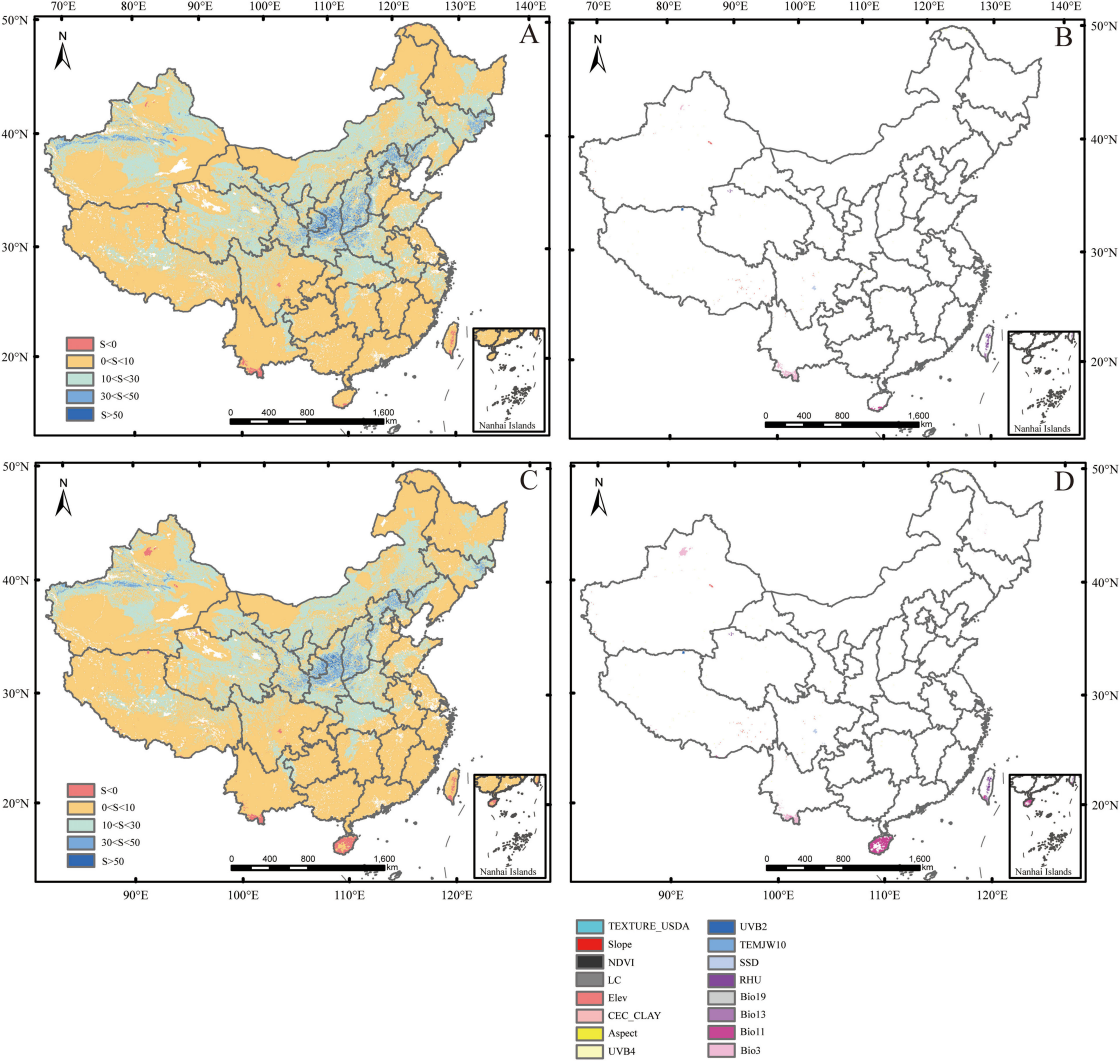
Multivariate environmental similarity surface (MESS) and the most dissimilar variable (MoD). **(A, C)** represent MESS for the periods 2081–2100 under SSP126 and SSP585 scenarios, respectively; **(B, D)** represent MoD for the periods 2081–2100 under SSP126 and SSP585 scenarios, respectively. Factor abbreviations were shown in **Table 1**.

The MESS is a spatial analysis method for quantifying environmental similarity. It is used to assess the multi-dimensional differences between future periods and the baseline period in the study area. The MoD is a derived indicator in the MESS analysis, used to identify the key variables that drive environmental differences. Under SSP126 and SSP585 scenarios, the average similarities were 8.76% and 7.85%, respectively, while the negative similarity percentages were 0.30% and 0.75%, respectively. SSP585 exhibited a greater degree of dissimilarity compared to SSP126, consistent with the higher radiative forcing associated with SSP585. This increased forcing is expected to amplify environmental deviations from historical conditions. Under both SSP126 and SSP585 scenarios, climate anomaly areas (S ≤ 0) within all predicted potential suitable habitats were primarily located in central Sichuan, southern Yunnan, Hainan, and central-southern Taiwan. The most dissimilar variables were Bio11, SSD, Bio3, RHU, and Bio13 (**Figure 7**).

## 4 Discussions

### 4.1 The key factors restricting the potential distribution

By scientifically regulating key factors restricting potential distribution for mango, it is beneficial to leap from “relying on the environment” to “precision agriculture” and lay the foundation for the sustainability of the tropical fruit industry.

In our study, two temperature factors (Bio11 and TEMJW10), a precipitation factor (Bio19), and a sunshine radiation factor (UVB2) significantly influenced the potential distribution of mango (**Table 1**), which aligns with mango’s growth condition preferences. High temperature, ample sunshine, and limited precipitation can promote its flowering, pollination, and fruit development, and appropriate heat accumulation is essential for the completion of the entire growth cycle. These factors directly shape the areas where mango can thrive, determining the boundaries of its potential distribution. For example, regions with consistently low temperatures, as limited by factors like Bio11 and TEMJW10, are less suitable for mango cultivation, as the cold inhibits the necessary physiological processes for growth. Similarly, areas with excessive or insufficient precipitation, as indicated by Bio19, may not support healthy mango growth, affecting the overall potential distribution map.

Previous studies have also highlighted the critical role of climate factors in plant distribution. For instance, Zhao et al. (2021) found that temperature factors had a greater impact than precipitation on Chinese fir distribution, with low temperatures restricting xylem cell production and high temperatures combined with low rainfall inhibiting growth. Trops (2023) reported that water scarcity in Spain led to a 70% decline in mango yield and smaller fruit size. Moreover, UV-B is closely related to CO2 concentration, nutrient availability, and water stress, and can induce morphological changes in plants (Zhang et al., 2018). Kang et al. (2025) emphasized that under high emission scenarios, shifts in temperature and precipitation patterns may profoundly affect the distribution of tropical plants, and extreme temperature events could reduce species range. These findings collectively underscore the significant influence of climate on plant population regeneration (Hansen et al., 2018).

Notably, some studies have put forward different viewpoints. Noordam (2024) indicated that topographic variables slowly affected by climate change, such as soil depth, soil texture, soil erodibility, elevation, and slope, could determine the potential distribution of mango. Zhao et al. (2021) demonstrated that under climate scenarios, climate anomaly in low-altitude areas would be related to temperature, while that in high-altitude areas would be related to precipitation. This indicated that altitude may affect Chinese fir’s potential distribution by modulating climate conditions. However, the topographic variables contributed minimally to our predictions. It was likely because temperature, precipitation, and solar radiation played more immediate and critical roles in shaping mango’s ecological adaptation and distribution. Moreover, Couet et al. (2022) demonstrated that species traits, such as dispersal ability and reproductive rate, could also influence distribution shifts, enabling species to colonize new suitable habitats more effectively. Gao (2021) proposed that mountain-adapted species may better withstand climate change by migrating upward to find suitable climatic conditions. Nevertheless, the influence of species traits on range and abundance shifts remains poorly understood. Different mango varieties exhibit distinct species traits, require unique optimal growth conditions, and occupy varied habitats. Therefore, considering these key factors, for mango plantations, in practical applications, a more accurate distribution of the potential suitable habitats should be obtained for different mango varieties to facilitate precise planting, harvesting, and management.

In response to the impact of these key factors, for mango plantations, several measures can be taken. To mitigate the increasing risks of extreme climate events (e.g., droughts and heavy rainfall) associated with climate change (Noordam, 2024; Chen and Sun, 2015), we proposed to: 1) Prioritize the development of water-saving irrigation and shade management; 2) Breed and promote drought-tolerant cultivars with enhanced resistance to humidity-induced diseases (e.g., anthracnose), but conduct rigorous pilot trials to evaluate the performance of drought/disease-resistant varieties under localized conditions before scaling up cultivation.

### 4.2 Analysis of the current potential distribution

The assessment indicates that the current potential suitable habitat for mango was mainly found in the tropical and subtropical climate zones of Hainan, Guangdong, Guangxi, Yunnan, southern Tibet, southern Sichuan, southern Guizhou, southeast Fujian, western Taiwan, and partial Chongqing (**Figure 3**). It is in agreement with the previous report, which indicated that mango was a fruit tree species widely distributed in all tropical and subtropical regions (Bally, 2006). The primary mango-producing regions in China were located in Yunnan, Guangxi, Hainan, Sichuan, Guangdong, Guizhou, Fujian, and Taiwan, with a few additional mango cultivation areas in Xizang (Guo et al., 2023). However, we have not found any records of mango distributed in Chongqing now. It indicated that parts of the current mango suitability areas simulated in this study were located in areas where they did not exist. This may be related to the niche conservation hypothesis. The core idea of this hypothesis is elaborated, which is that species tend to retain the niche characteristics of their ancestors. During the diffusion process, they will be restricted by their own physiological and ecological characteristics as well as geographical barriers, resulting in a lag between the actual distribution of species and the potential suitable distribution. Combined with the geographical environment and climatic conditions of Chongqing, it is analyzed that although there are simulated areas suitable for mango growth in this region, due to niche conservation, the diffusion of mango may be affected by factors such as mountain barriers and lack of transmission media, leading to the absence of actual planting records (Wiens and Graham, 2005). In addition, this may be due to limitations imposed by the presence and interactions of other species, resulting in the basic ecological niche of mango being greater than its realized ecological niche (Hill et al., 2024; Pearson and Dawson, 2003). Overall, mango cultivation can be expanded to Chongqing in the future.

### 4.3 Potential distribution changes in the future

Analyzing the potential suitable habitats for mango under climate scenarios can provide a theoretical basis for the rational planning of mango cultivation in southern China, thereby enhancing agricultural productivity and planning standards.

There are three possible responses of species to climate change: range shift, adaptation, and extinction (Couet et al., 2022). Many studies demonstrated that the distribution ranges of species could shift northward or to higher latitudes due to global warming (Couet et al., 2022). In the results from Zhao et al. (2021), under SSP126 and SSP585 scenarios, the potential suitable habitats for Chinese fir showed a tendency to migrate to high-altitude regions. Zhang et al. (2018) pointed out that, with global warming, a shift in potential distribution for two peony species to higher elevations would become more significant. Kang et al. (2025) found that the potential suitable habitat for *Alpinia officinarum* shifted northwestward, more markedly under high emission scenarios (SSP370, SSP585). The temperature rose more sharply in high-altitude regions than in low-altitude regions, making this phenomenon particularly evident there (Zhao et al., 2021). Similarly, in our study, there was a trend for mango to shift toward higher altitude and latitude under SSP585 scenario, whereas there was no discernible trend under SSP126 scenario (**Figure 3**). Under SSP585 scenario, high emissions drive substantial warming, compelling species to shift to cooler (higher altitudes/latitudes) areas to track their climatic niche. On the contrary, under SSP126 scenario, strict emission reduction policies effectively mitigate the magnitude of climate change, resulting in a relatively slow and stable temperature rise, and less environmental pressure on species, thus requiring no significant changes in their distribution range, leading to unclear migration trends. This may be the main reason for the differences in changes of potential suitable habitats for mango under the two climate scenarios.

Certain species may adapt to climate warming by accelerating phenology and extending the growing season (Zhang et al., 2018). Excessive warming could harm plants, especially narrow-ranged species, which are more vulnerable than widespread ones due to limited climate adaptability. For example, climate change differentially affected two peony species: their suitable habitats expanded under the RCP2.6 scenario while showing contrasting responses (one increased, one decreased) under the RCP8.5 scenario. This variation stemmed from their dormancy-breaking temperature optimum (10-15°C), as excessive warming inhibited seed regeneration (Zhang et al., 2018). Kang et al. (2025) found that *Alpinia officinarum*’s potential suitable habitats expanded under low emission scenarios (SSP126, SSP245) but sharply declined under high emission scenarios (SSP370, SSP585), shrinking its range by approximately 3.7% and 19.8%, respectively. These studies illustrated the expansion of species distribution under low-emission scenarios and the contraction of species distribution under high-emission scenarios. However, our projections demonstrated that the potential suitable habitats for mango under both SSP126 and SSP585 scenarios would expand (**Tables 2**, **3**). This was consistent with the findings of some scholars. Noordam (2024) projected an expansion of potential distribution for mango cultivation in southern Europe under both SSP126 and SSP585 scenarios. Gao (2021) observed increased areas of potential distribution for mango under both RCP 4.5 and RCP 8.5 scenarios, with a more pronounced expansion under the higher emission scenario (RCP 8.5). These suggest strong adaptability to forthcoming climate conditions, including high-emission pathways.

Under SSP126 and SSP585 scenarios, the main climate anomaly areas were located in Hainan, central Sichuan, southern Yunnan, and central southern Taiwan (**Figures 7A, C**). Among them, under two climate scenarios, the potential suitable habitats in the Sichuan-Chongqing area, including central Sichuan, were predicted to significantly expand, while other areas were still covered by the current potential suitable habitats (**Figures 5B, D, F**; **6B, D, F**). This indicated that mango would have strong adaptability under climate change conditions in China. In addition, SSD was the most dissimilar variable in central Sichuan (**Figures 7B, D**). This indicated that SSD in central China, such as the Sichuan-Chongqing area, would have a positive effect on mango growth in the future.

Given the projected shifts in suitable areas, we recommend expanding mango cultivation into higher-latitude and higher-elevation regions, particularly in central southern China (e.g., Sichuan, Chongqing), while adopting adaptive management and dynamic monitoring to reduce climate risks.

## 5 Conclusions

Based on the Maxent model, the potential suitable habitats for mango in China were predicted. According to the assessment, the current potential suitable habitats covered approximately 4.81×105 km², primarily located in the southern provinces of China, within the tropical and subtropical regions. Under climate scenarios of both SSP585 and SSP126, the projected potential suitable habitats for mango exhibited significant expansion compared to the current distributions. The potential suitable habitats not only encompassed the southern provinces of China that were already covered but also extended northward to include central provinces, particularly Sichuan and Chongqing. Under SSP585 scenario, the degree of climate anomaly was higher, and mango tended to transfer to high-altitude/latitude areas. However, the degree of climate anomaly was lower, and the trend of mango transferring to high-altitude/latitude areas was not obvious under SSP126 scenario. The jackknife test showed that Mean Temperature of Coldest Quarter, Annual accumulated temperature (≥10°C), Precipitation of Coldest Quarter, and UV-B Seasonality were the main factors affecting the distribution of the potential suitable habitats for mango. Their cumulative contribution rate and permutation importance reached values as high as 83.8% and 92.6%, respectively. Therefore, we suggest strategically expanding mango cultivation to high-latitude/altitude areas in the future, especially in central China, including Sichuan and Chongqing. In addition, to cope with the drought and rainstorm risks brought by climate change, we suggest that in practical application: 1) Giving priority to the development of water-saving irrigation systems and shading management; 2) Obtaining the potential suitable habitats distribution for different varieties of mango for precise management, while breeding and promoting drought-resistant varieties with stronger resistance to humidity-induced diseases such as anthracnose.

## Data Availability

Publicly available datasets were analyzed in this study. This data can be found here: The 19 bioclimatic factors (2.5 min) were obtained from the World Climate Database (Fick and Hijmans, 2017). The altitude, slope, and aspect were extracted from the Digital Elevation Model (25 m), which was obtained from the Geospatial Data Cloud (http://www.gscloud.cn/). The indices of land cover (15 sec) were obtained from the Global Map data archives (https://globalmaps.github.io/). The 16 soil factors (0-20 cm depth layers) were obtained from Harmonized World Soil Database (https://www.fao.org/soils-portal/data-hub/soil-maps-and-databases/harmonized-world-soil-database-v20/en/). The Global UV-B radiation factors (UVB1-6, 15 min) were obtained from the gIUV database (https://www.ufz.de/gluv/index.php?en=32435). The Normalized Difference Vegetation Indices (30 sec) downloaded from Geographical Information Monitoring Cloud Platform (http://www.dsac.cn/), were obtained by taking the average monthly values from 1970 to 2000. In addition, annual sunshine duration, annual relative humidity, and annual accumulated temperature (≥ 10°C, ≥ 5°C and ≥ 0°C) downloaded from the Resource and Environmental Science Data Platform (1 km, https://www.resdc.cn/DOI/DOI.aspx?DOIID=96), were obtained by taking their average values from 1970s to 2000s.
